# Supplementary material for: Two-stage binding of mitochondrial ferredoxin-2 to the core iron-sulfur cluster assembly complex
Source: Nat Commun. 2024 Dec 4;15:10559. doi: 10.1038/s41467-024-54585-4 (PMC11618653; doi:10.1038/s41467-024-54585-4)
Supplement: Supplementary file 1 — Supplementary Information [file 41467_2024_54585_MOESM1_ESM.pdf]

Supplementary Information for

Two-stage binding of mitochondrial ferredoxin-2 to the core iron-sulfur cluster assembly complex

Ralf Steinhilper, Linda Boß, Sven-A. Freibert, Vinzent Schulz, Nils Krapoth, Susann Kaltwasser, Roland Lill*, Bonnie J. Murphy*

*Corresponding authors. Email: Bonnie J. Murphy, bonnie.murphy@biophys.mpg.de;
Roland Lill, lill@staff.uni-marburg.de

The PDF file includes:

Supplementary Figures 1-14
Supplementary Tables 1-4
Supplementary References

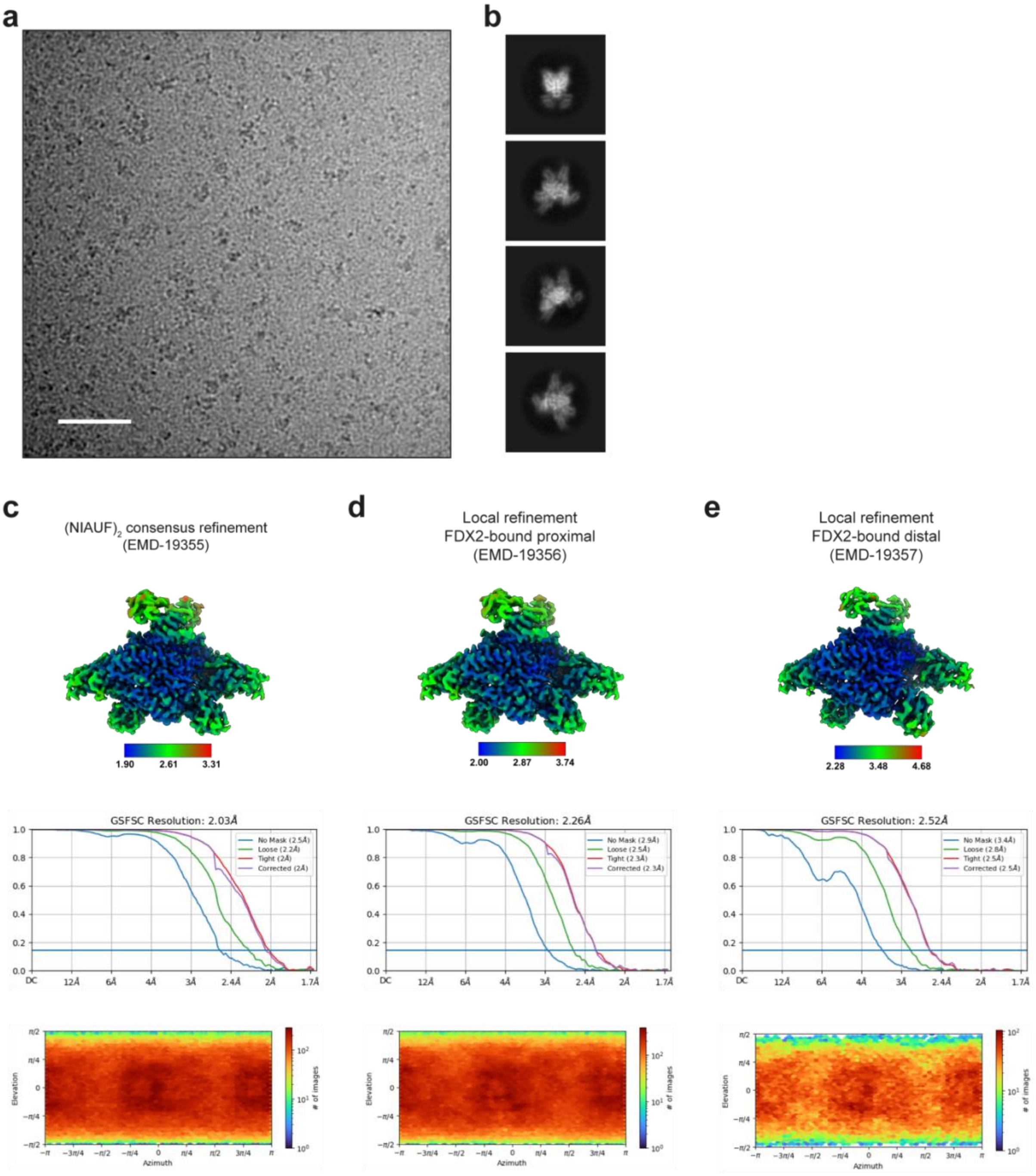

Supplementary Fig. 1: Cryo-EM data processing of the $(NIAUF)_2$ dataset. (**a**) Representative cryo-EM micrograph (scale bar 40 nm), (**b**) representative 2D class averages. (**c-e**) Local resolution estimation (0.5 FSC criterion), Fourier Shell Correlation (FSC) curves with global resolution (0.143 FSC criterion) and angular distribution for (c) the $(NIAUF)_2$ consensus refinement (EMD-19355), (d) the local refinement of the FDX2-bound proximal state (EMD-19356), and (e) the local refinement of the FDX2-bound distal state (EMD-19357).

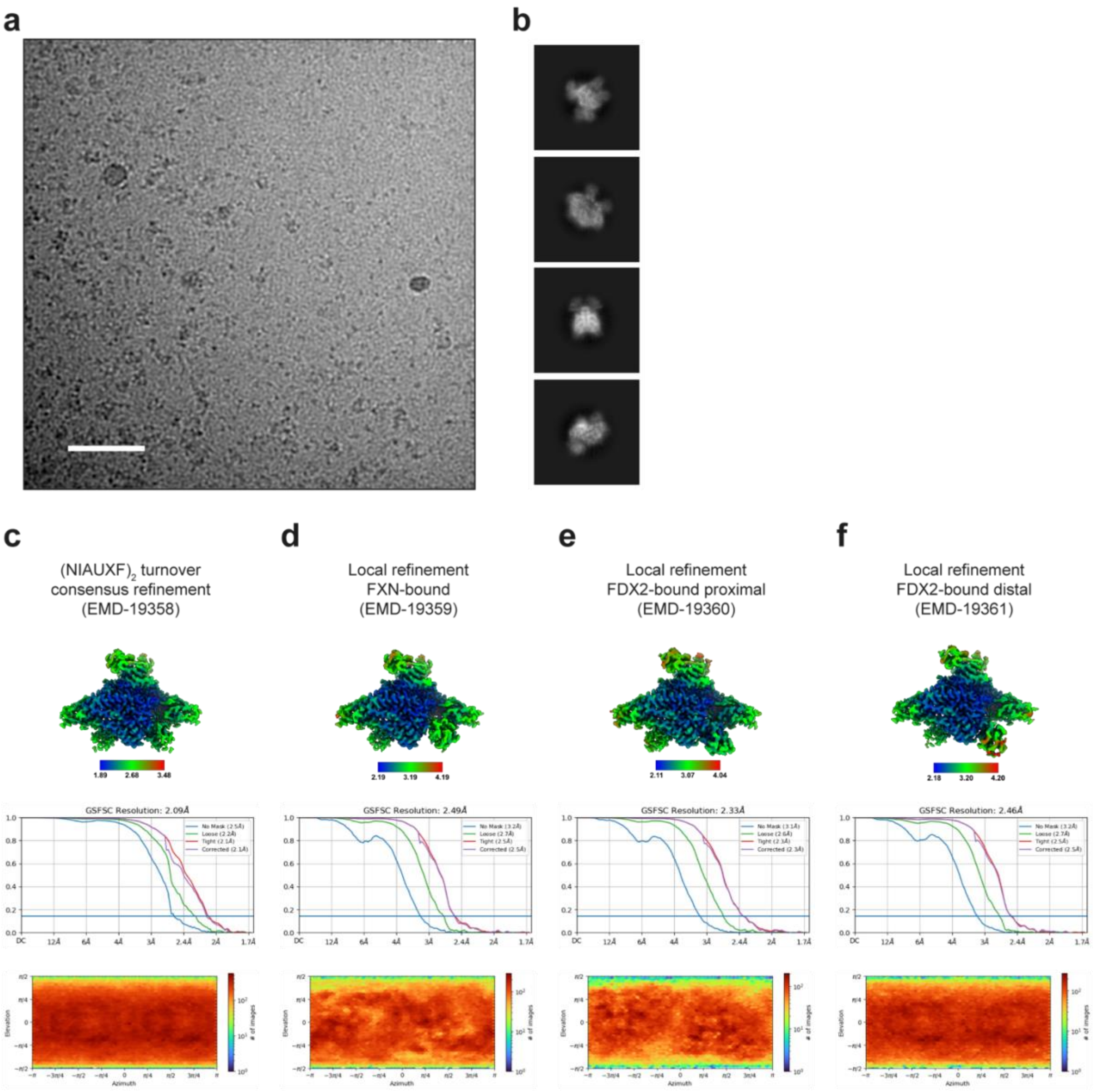

Supplementary Fig. 2: Cryo-EM data processing of the (NIAUXF)$_2$ turnover dataset. (**a**) Representative cryo-EM micrograph (scale bar 40 nm), (**b**) representative 2D class averages, **(c-f)** Local resolution estimation (0.5 FSC criterion), Fourier Shell Correlation (FSC) curves with global resolution (0.143 FSC criterion) and angular distribution for (c) the (NIAUXF)$_2$ turnover consensus refinement (EMD-19358), (d) local refinement of the FXN-bound class (EMD-19359), (e) local refinement of the FDX2-bound proximal state (EMD-19360), (f) local refinement of the FDX2-bound distal state (EMD-19361).

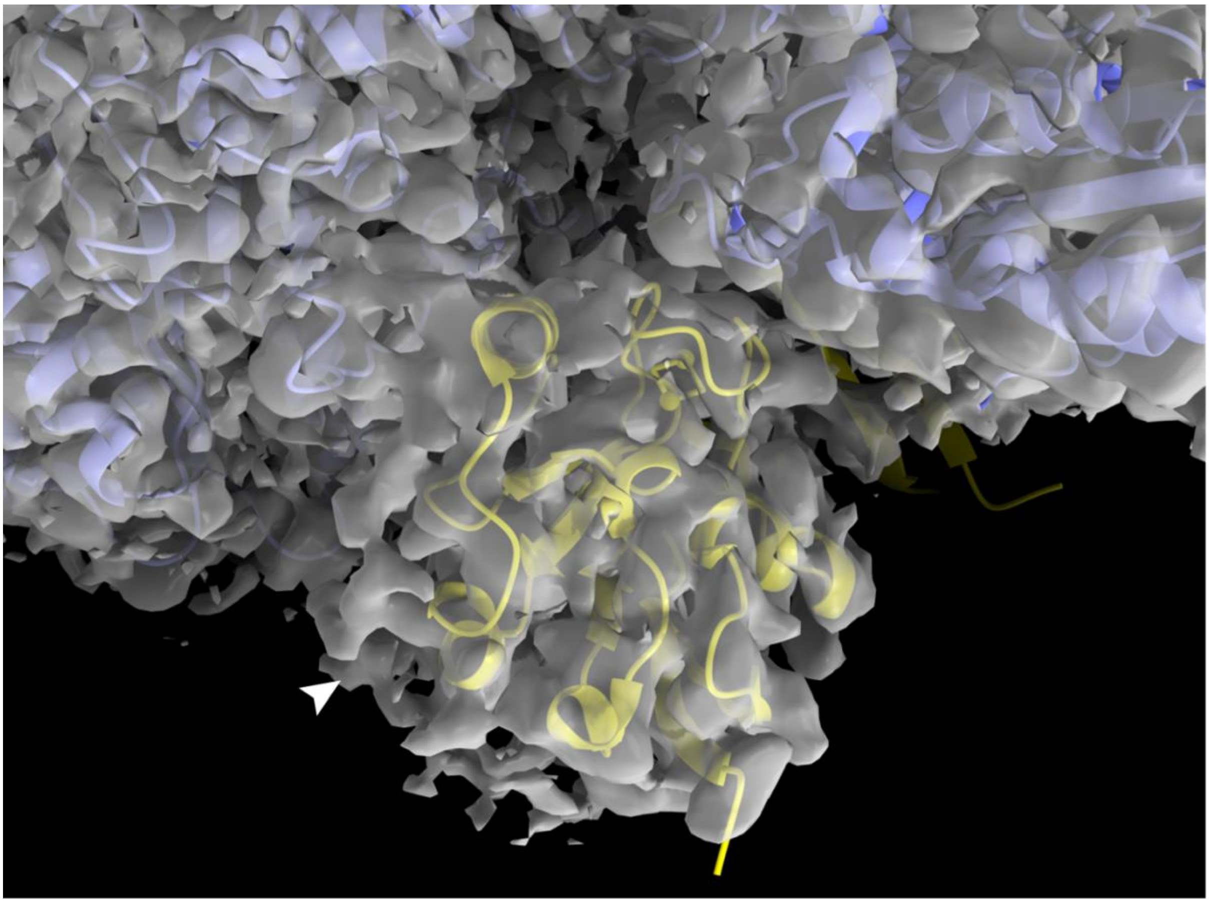

Supplementary Fig. 3: FDX2-binding region in the $(NIAUF)_2$ consensus refinement map. The atomic model of the FXN-bound core ISC complex ($(NIAUX)_2$, PDB 6NZU; purple), from which FXN was deleted, and the model of FDX2 (PDB 2Y5C; lemon) were rigid-body fitted into the consensus refinement map. Unmodelled density in the map (arrow) is due to heterogeneity in the position of FDX2 binding.

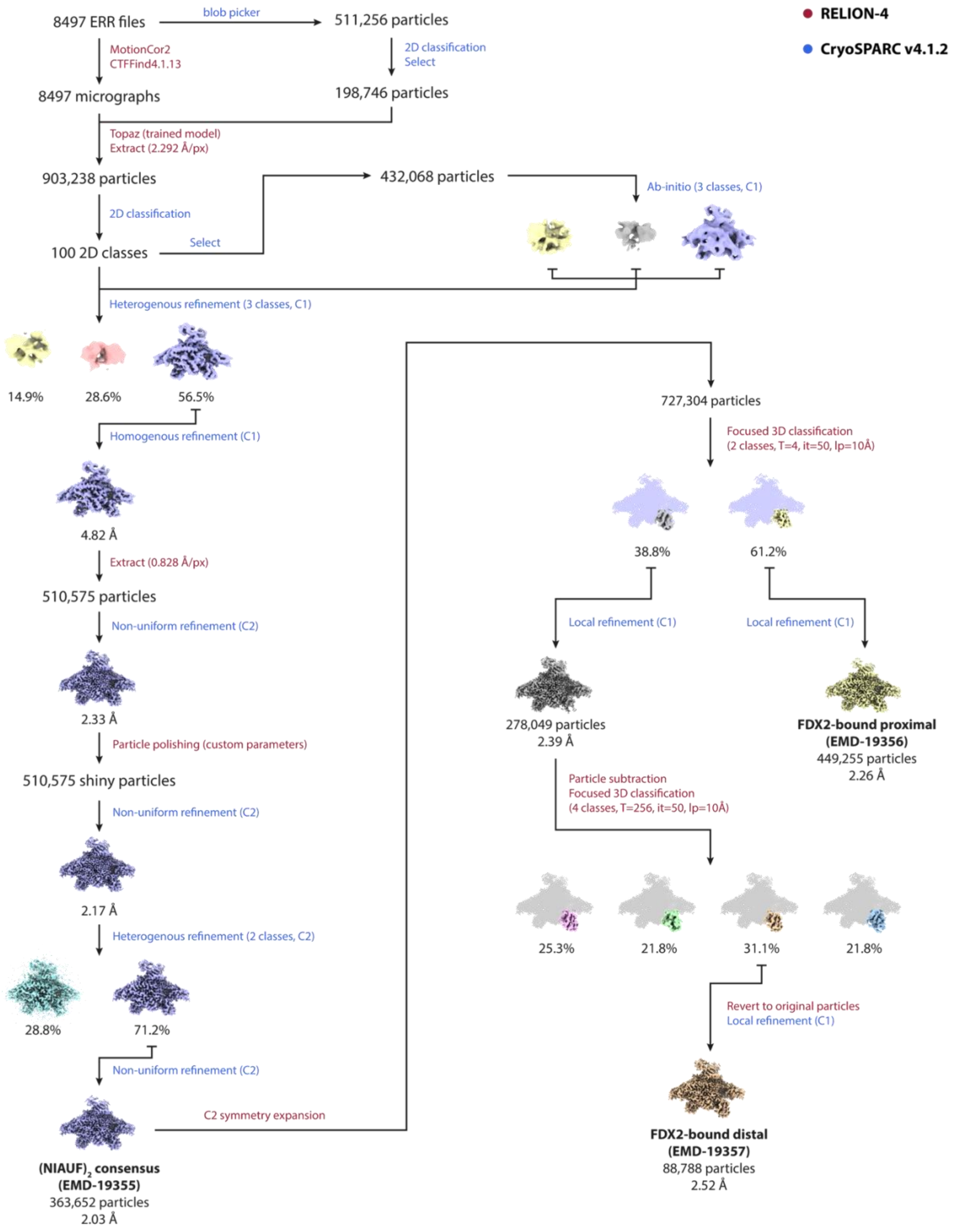

Supplementary Fig. 4: Data processing scheme for the $(NIAUF)_2$ dataset. Processing steps were performed in RELION-4 (red) and CryoSPARC v4.1.2 (blue).

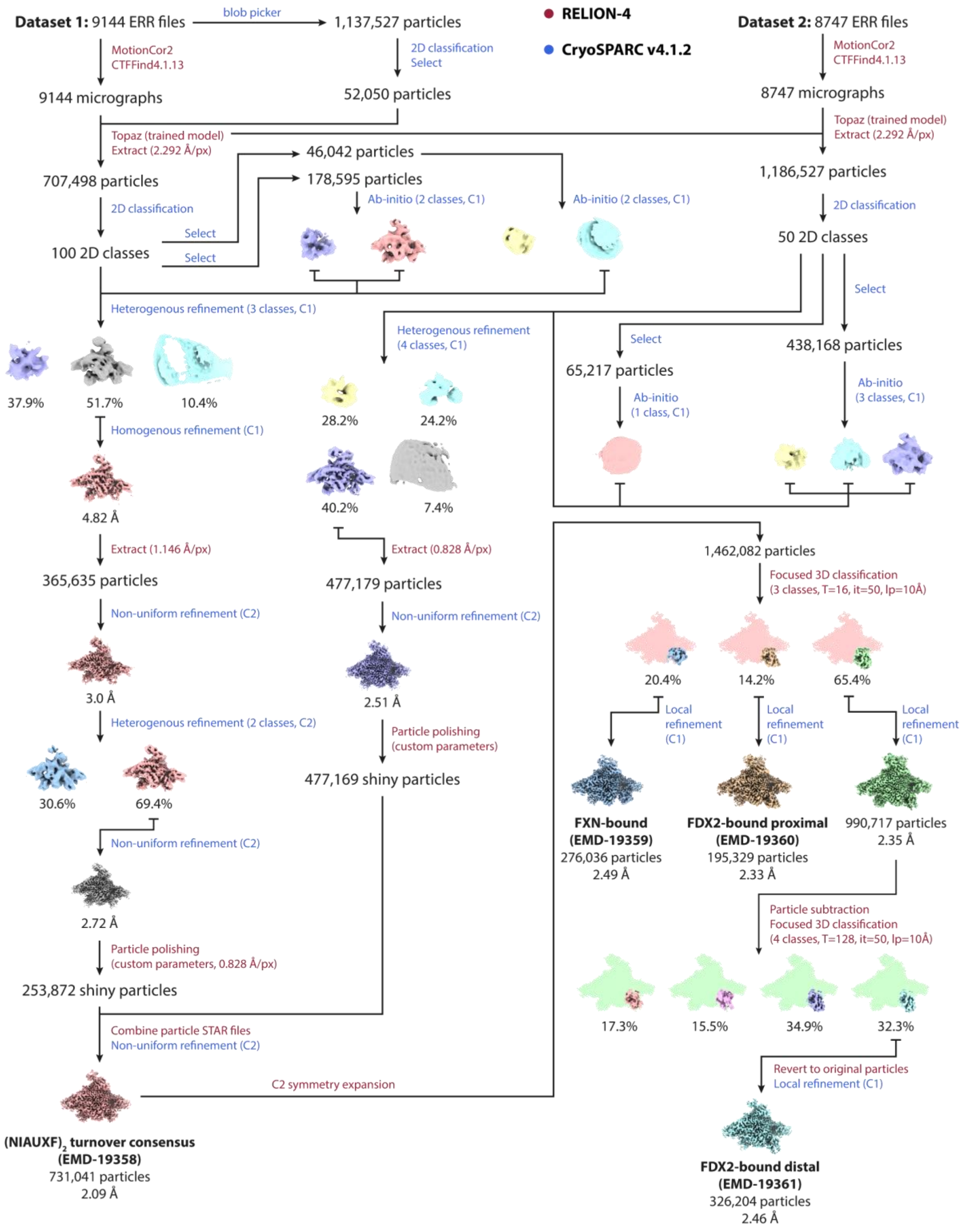

Supplementary Fig. 5: Data processing scheme for the (NIAUXF)$_2$ turnover datasets. Processing steps were performed in RELION-4 (red) and CryoSPARC v4.1.2 (blue).

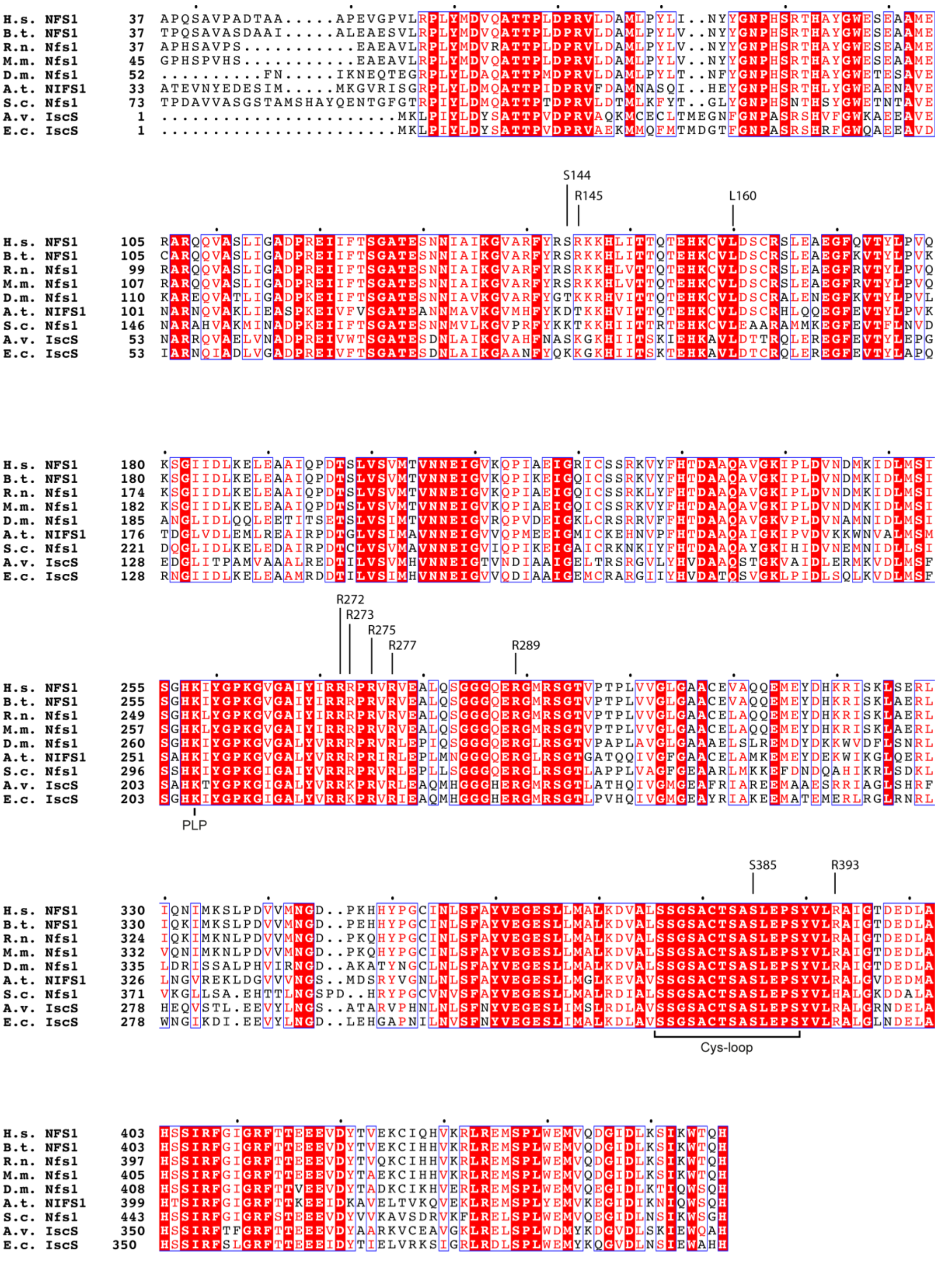
H.s. NFS1
B.t. NFS1
R.n. Nfs1
M.m. Nfs1
D.m. Nfs1
A.t. NIFS1
S.c. Nfs1
A.v. IscS
E.c. IscS
S144
R145
L160
R272
R273
R275
R277
R289
PLP
S385
R393
Cys-loop

Supplementary Fig. 6: Multi-sequence alignment of NFS1-like proteins. The Cys-loop region, the PLP cofactor linked to Lys258 and residues interacting with human FDX2 are annotated. Sequence identifiers: *H. sapiens* NFS1 (Q9Y697), *B. taurus* NFS1 (A5PKG4), *R. norvegicus* Nfs1 (Q99P39), *M. musculus* Nfs1 (Q9Z1J3), *D. melanogaster* Nfs1 (Q9VKD3), *A. thaliana* NIFS1 (O49543), *S. cerevisiae* Nfs1 (P25374), *A. vinelandii* IscS (O31269), *E. coli* IscS (P0A6B7).

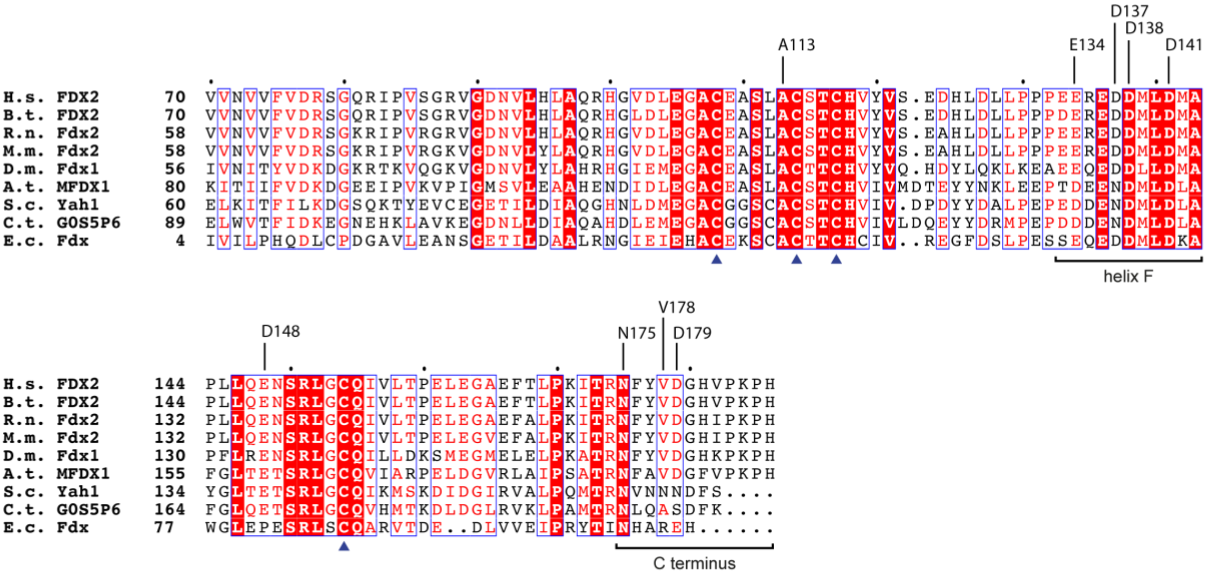

Supplementary Fig. 7: Multi-sequence alignment of FDX2-like proteins. Residues interacting with NFS1/NFS1’ are annotated. Blue triangles indicate the [2Fe-2S] cluster-coordinating cysteines Cys108, Cys114, Cys117 and Cys154 of human FDX2. Sequence identifiers: *H. sapiens* FDX2 (Q6P4F2), *B. taurus* FDX2 (Q05B51), *R. norvegicus* Fdx2 (D4A8N2), *M. musculus* Fdx2 (Q9CPW2), *D. melanogaster* Fdx1 (P37193), *A. thaliana* MFDX1 (Q9M0V0), *S. cerevisiae* Yah1 (Q12184), *C. thermophilum* Putative 2 iron, 2 sulfur cluster binding protein (G0S5P6), *E. coli* Fdx (P0A9R4).

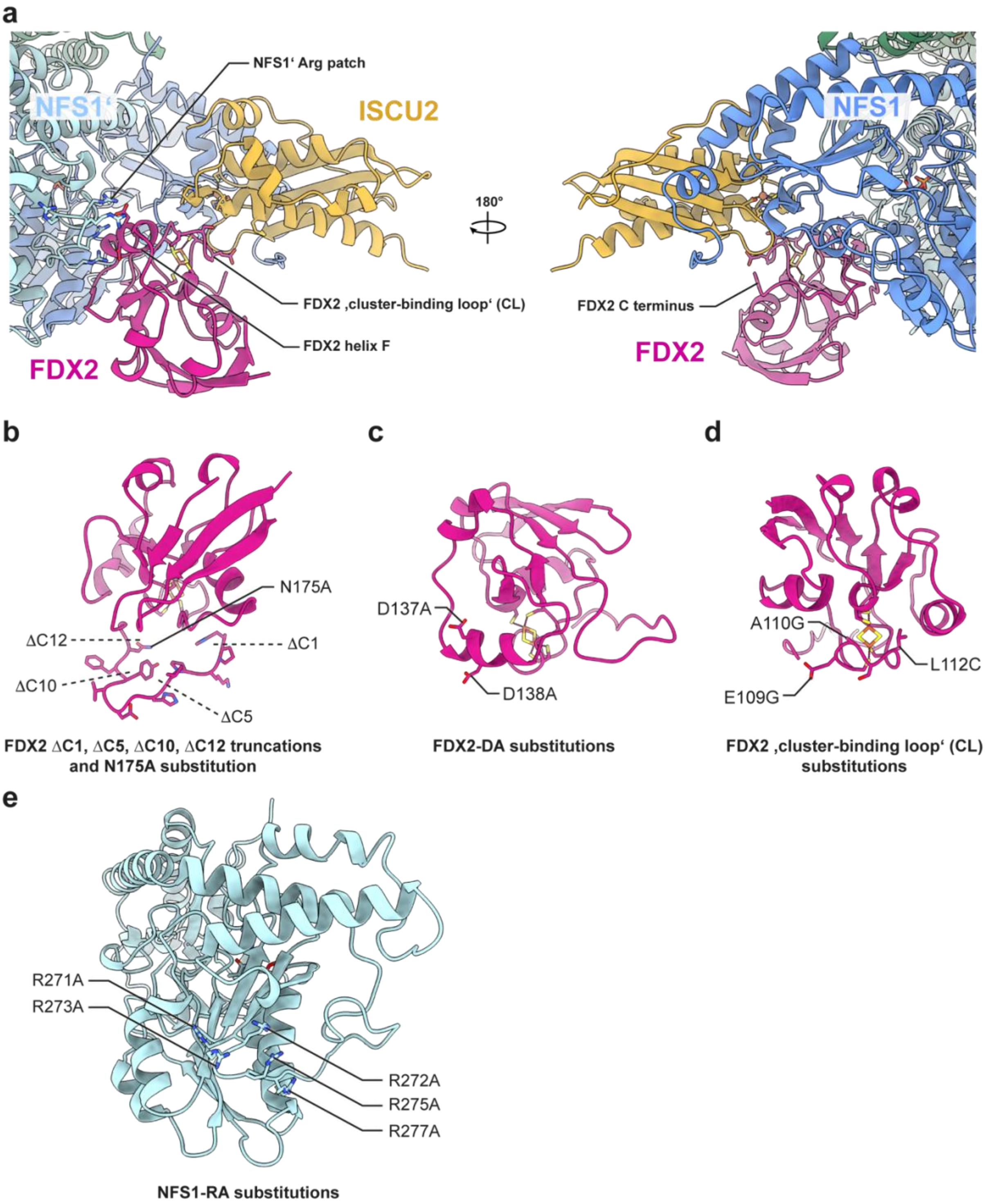

Supplementary Fig. 8: Variants for testing the contact areas between FDX2 and NFS1 or ISCU2 within the core ISC complex. (**a**) 3D overview of the positions altered in FDX2 or NFS1. (**b-d**) Exact truncations or amino acid exchanges at the C terminus (b), at the salt bridge in helix F (c), and at the ‘cluster-binding loop’ (CL) of FDX2 (d). (**e**) Amino acid exchanges at the Arg patch of NFS1.

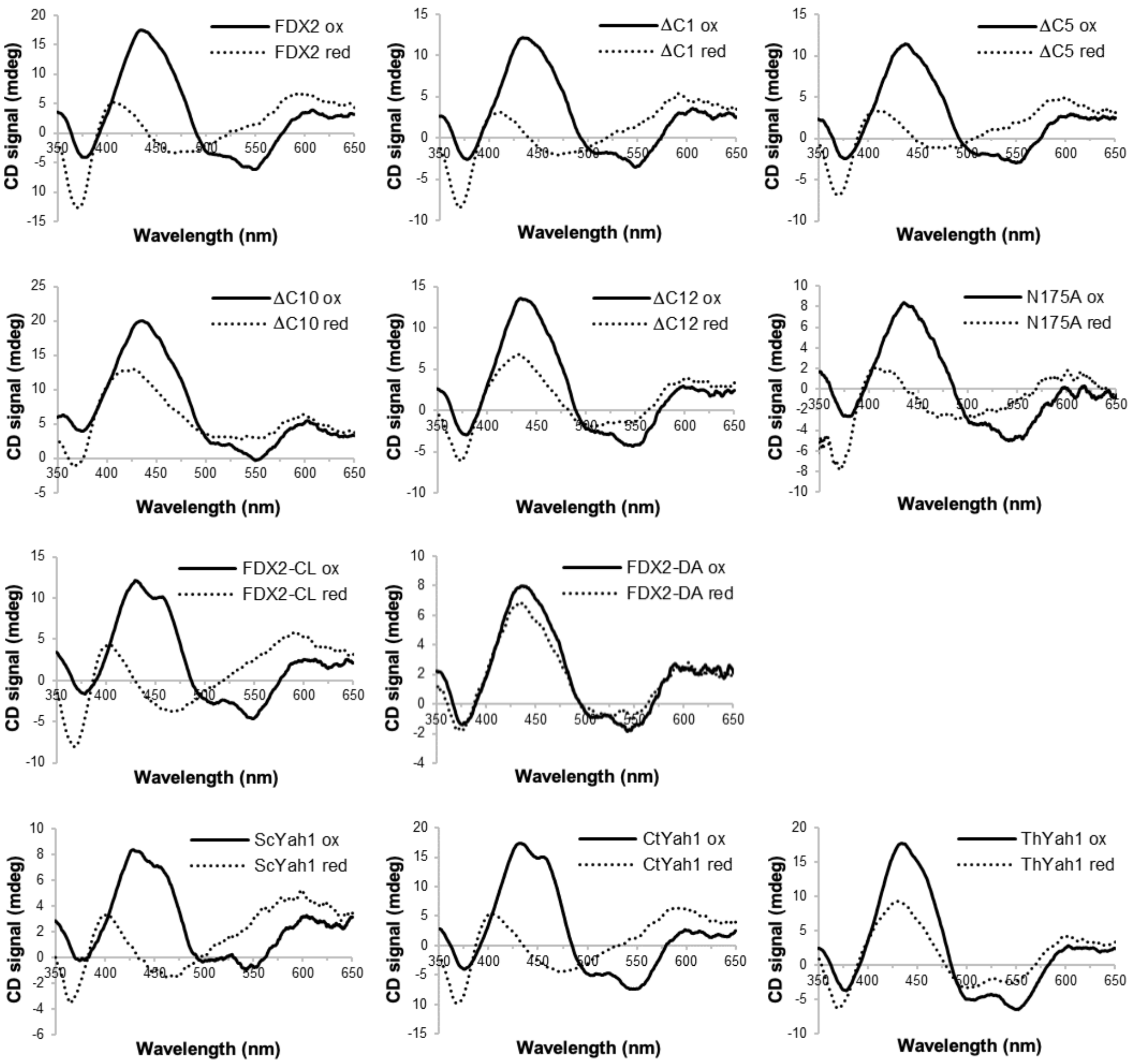

Supplementary Fig. 9: Circular dichroism spectroscopy of human FDX2, its variants, and fungal ferredoxins. CD spectra were recorded under anaerobic conditions for as-isolated (ox) and NADPH-FDXR-reduced (red) FDX2 and its variants (20 µM each). Reduction was performed anaerobically under conditions of the standard [2Fe-2S] cluster synthesis assay using NADPH and FDXR (see Methods). FDX2-DA cannot be reduced under these conditions. Source data are provided as a Source Data file.

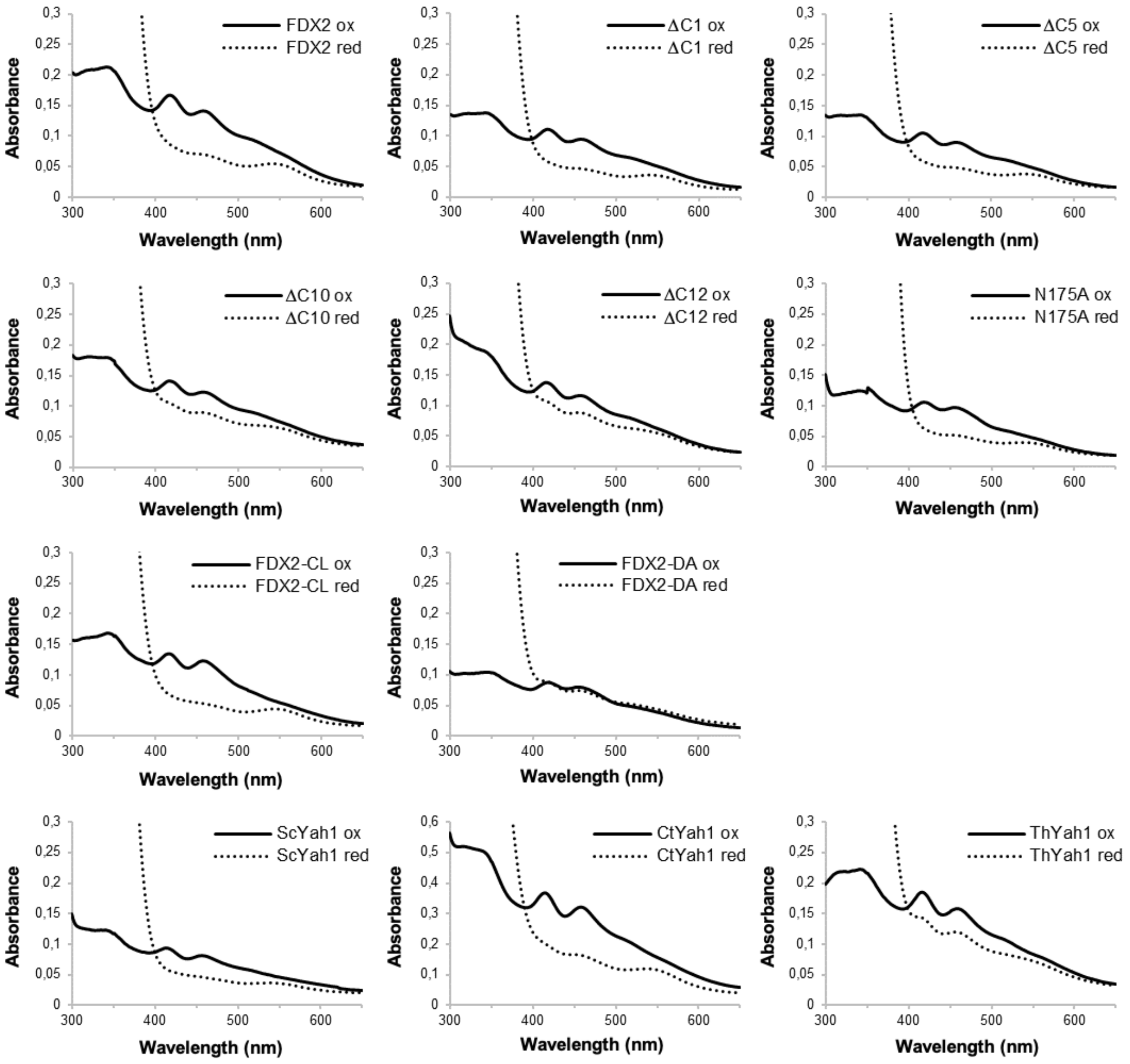

Supplementary Fig. 10: UV-Vis spectroscopy of human FDX2, its variants, and fungal ferredoxins. UV/Vis spectra of the proteins from Supplementary Fig. 9 were recorded. The oxidized [2Fe-2S] cluster is indicated by characteristic absorption peaks at 420 and 460 nm which are almost absent upon physiological reduction by NADPH and FDXR. FDX2-DA cannot be reduced under these conditions. Source data are provided as a Source Data file.

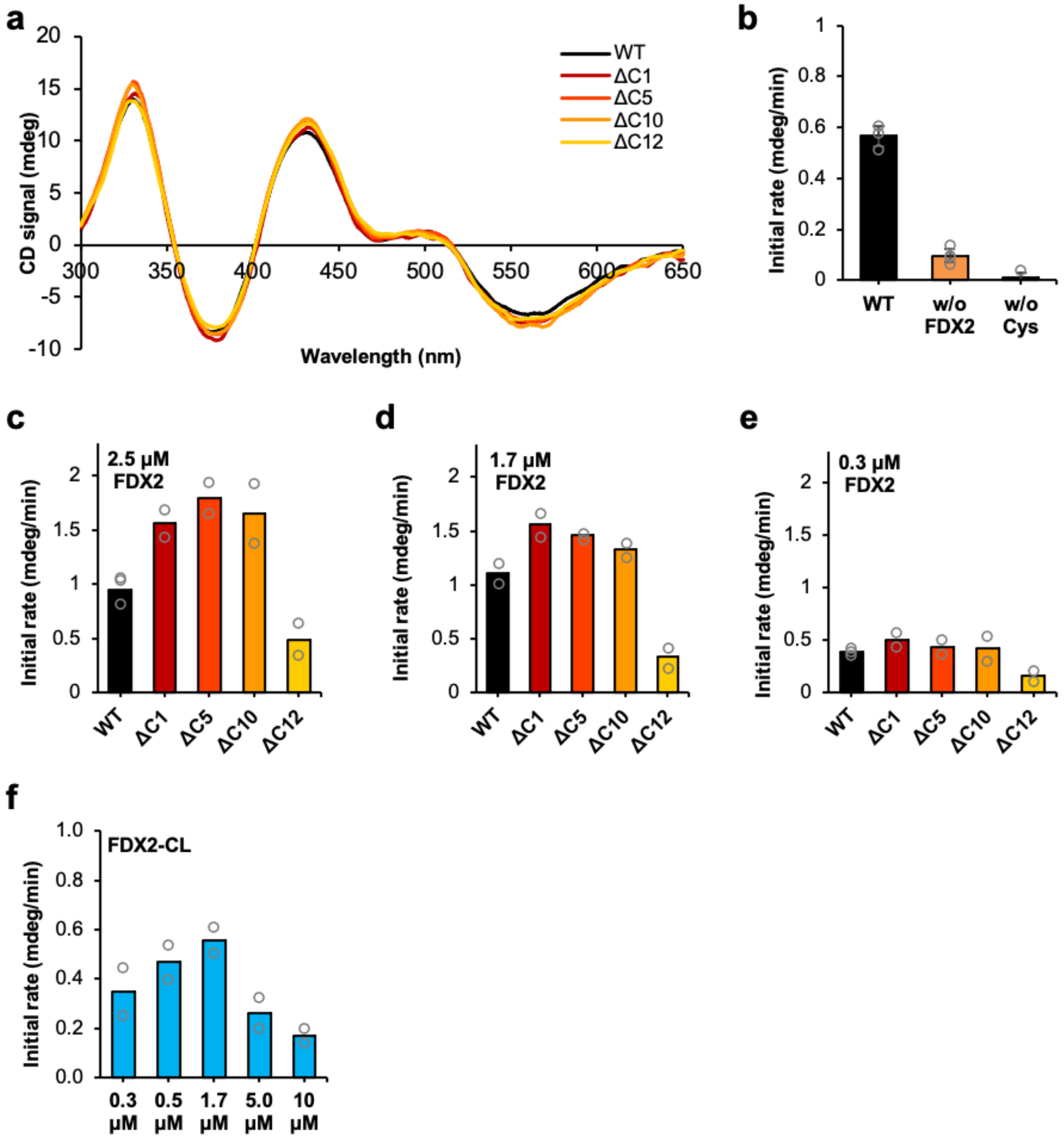

Supplementary Fig. 11: Enzymatic *in vitro* [2Fe-2S] cluster synthesis with different FDX2 variants. (related to Fig. 5). (**a**) CD spectra were recorded anaerobically after completion of [2Fe-2S] cluster synthesis on ISCU2 by wild-type (WT) FDX2 or its indicated C-terminal variants as described in Fig. 5b. For all FDX2 proteins, the [2Fe-2S] cluster end product on ISCU2 was similar. (**b**) Initial rates of [2Fe-2S] cluster synthesis reactions with or without (w/o) FDX2 or without addition of Cys under standard conditions (5 µM FDX2). Values are the mean ±SD (for w/o FDX2: n=4; for WT, w/o Cys: n=3; technical replicates). (**c-e**) Comparison of initial rates of cluster synthesis for the indicated concentrations of WT FDX2 or its variants (for WT in (c) and (e): n=3, for others: n=2). (**f**) Initial rates of [2Fe-2S] cluster synthesis reactions with the indicated FDX2-CL concentrations (n=2; related to Fig. 6c). Source data are provided as a Source Data file.

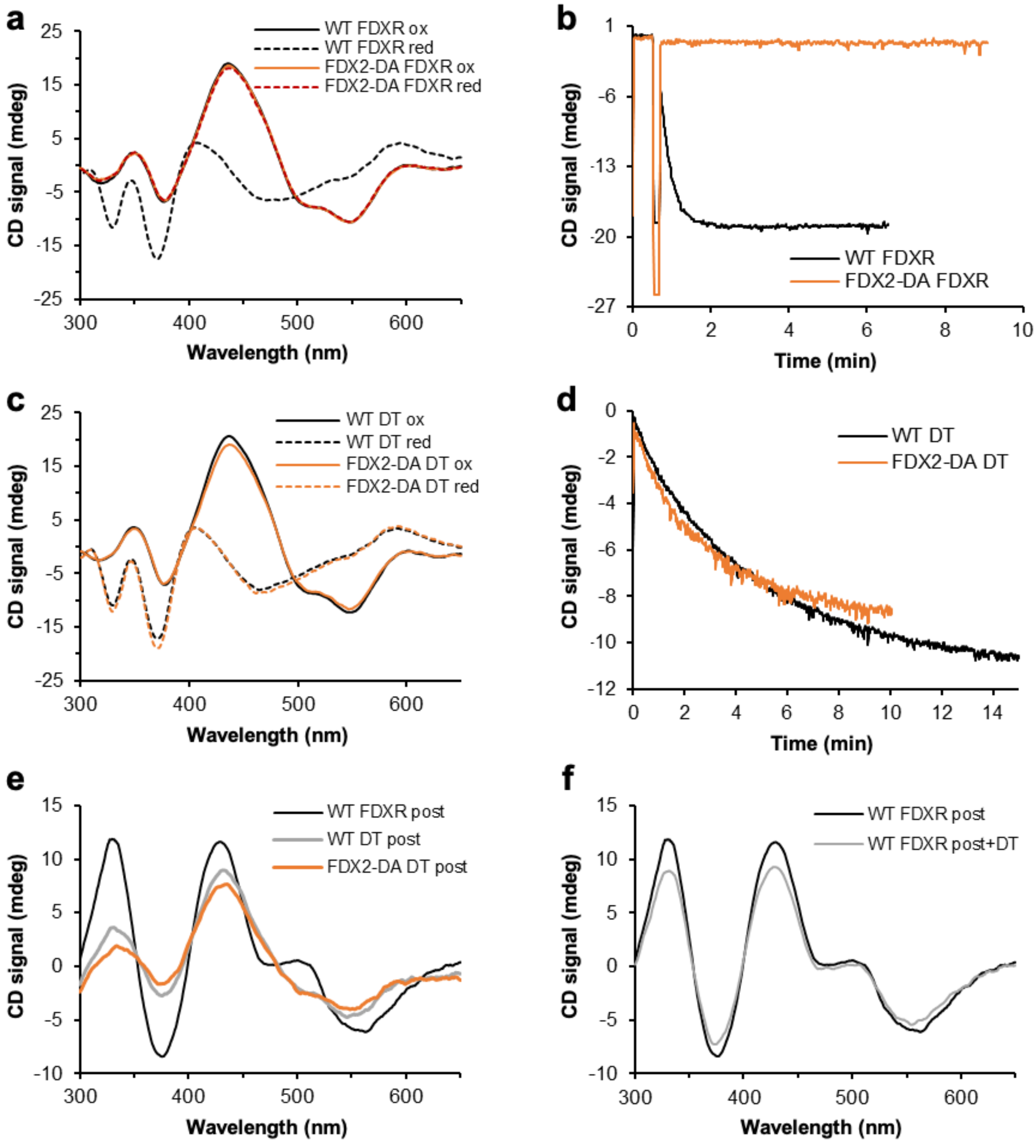

Supplementary Fig. 12: Enzymatic *in vitro* [2Fe-2S] cluster synthesis with the FDX2-DA variant after reduction with dithionite. (related to Fig. 6). (**a**) CD spectra of wild-type (WT) FDX2 and the FDX2-DA variant (20 µM each) either as isolated (ox) or after anaerobic reduction with 0.5 mM NADPH and 0.8 µM FDXR (see Methods). FDX2-DA cannot be reduced under these conditions. (**b**) Time-resolved CD signal changes after addition of NADPH-FDXR to FDX2 or FDX2-DA as described in part (a). (**c**) CD spectra of as isolated (ox) proteins from (a) and after anaerobic reduction with 1.6 mM sodium dithionite (DT, red). Both proteins can be reduced under these conditions. (**d**) Time-resolved CD signal changes after addition of DT to FDX2 or FDX2-DA. (**e**) CD spectra of samples from Fig. 6d recorded 20 min after reaction start. (**f**) A standard enzymatic [2Fe-2S] cluster synthesis reaction with NADPH-FDXR was performed, and a CD spectrum was recorded. The sample was then treated with 0.8 mM DT and after 10 min another CD spectrum was recorded. Apart from some bleaching no spectral changes were observed. Source data are provided as a Source Data file.

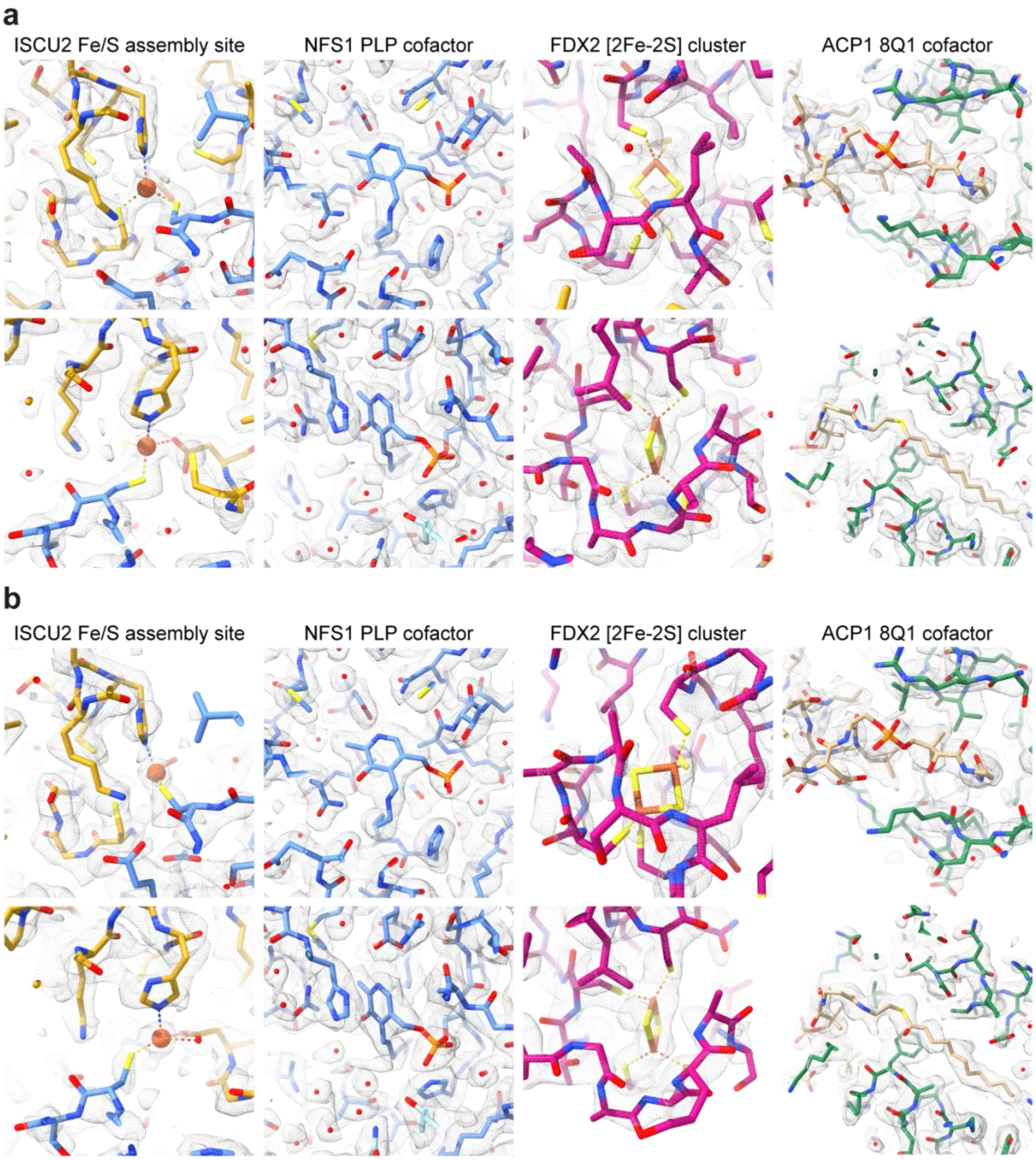

Supplementary Fig. 13: Atomic model and map showing key cofactors for structures obtained from the $(NIAUF)_2$ dataset. a, $(NIAUF)_2$ FDX2-bound proximal state (PDB 8RMC; EMD-19356). **b**, $(NIAUF)_2$ FDX2-bound distal state (PDB 8RMD; EMD-19357).

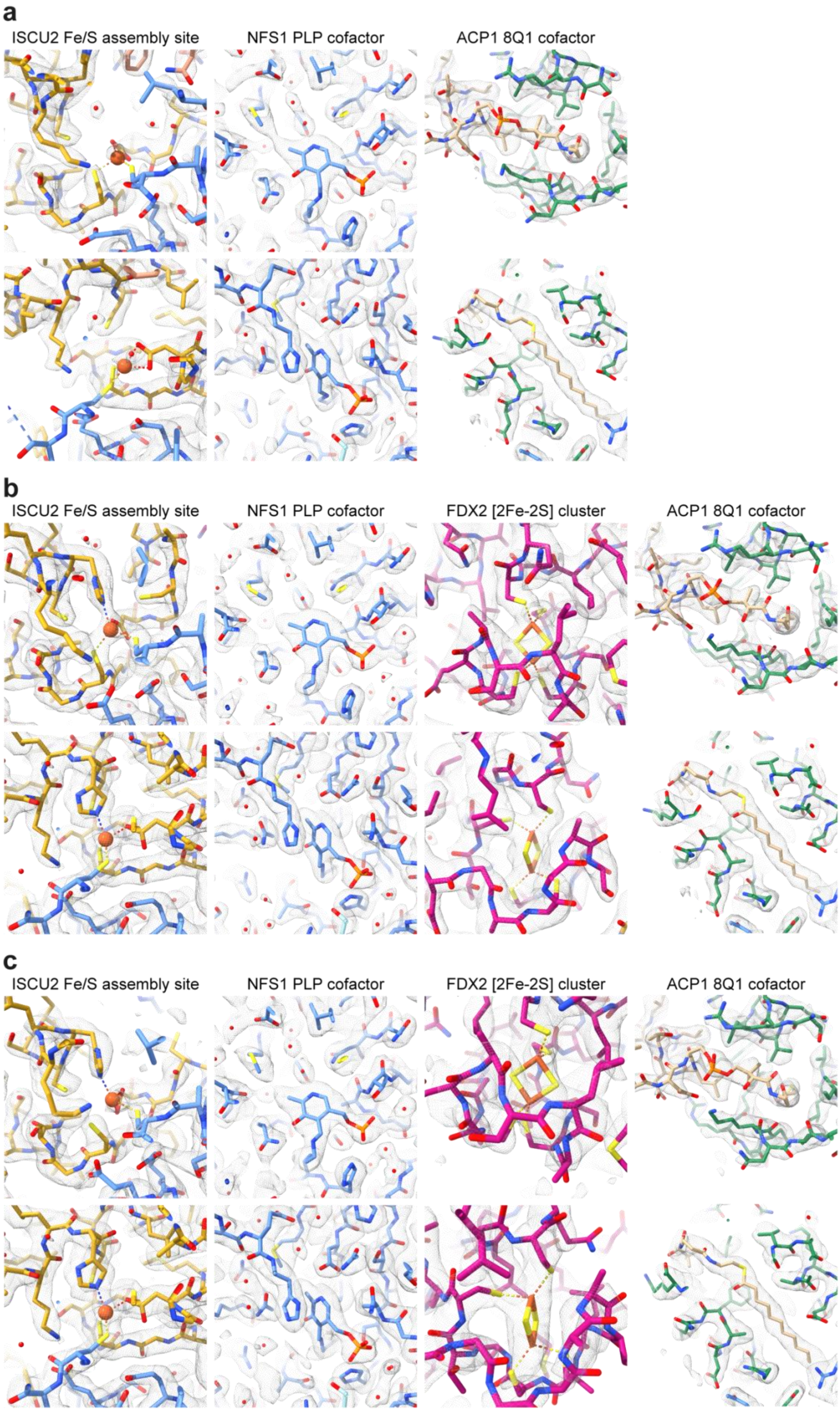

a
ISCU2 Fe/S assembly site
NFS1 PLP cofactor
ACP1 8Q1 cofactor
b
ISCU2 Fe/S assembly site
NFS1 PLP cofactor
FDX2 [2Fe-2S] cluster
ACP1 8Q1 cofactor
c
ISCU2 Fe/S assembly site
NFS1 PLP cofactor
FDX2 [2Fe-2S] cluster
ACP1 8Q1 cofactor

Supplementary Fig. 14: Atomic model and map showing key cofactors for structures obtained from the $(NIAUXF)_2$ turnover dataset. **a**, $(NIAUXF)_2$ turnover, FXN-bound state (PDB 8RME; EMD-19359). **b**, $(NIAUXF)_2$ turnover, FDX2-bound proximal state (PDB 8RMF; EMD-19360). **c**, $(NIAUXF)_2$ turnover, FDX2-bound distal state (PDB 8RMG; EMD-19361).

Supplementary Table 1: Protein sequences. Residues in brackets were removed by proteolytic cleavage and are not present in the final sample preparation.

Protein	Sequence	Uniprot ID
NFS1	MSLRPLYMDVQATTPLDPRVLDAMLPYLINYYGNPHSRTHAYGWESEAAMERARQQVASLIGADPREIIFTSGATESNNIAIKGVARFYRSRKKHLITTQTEHKCVLDSCRSLEAEGFQVTYLPVQKSGIIDLKELEAAIQPDTSLVSVMTVNNEIGVKQPIAEIGRICSSRKVYFHTDAAQAVGKIPLDVNDMKIDLMSISGHKIYGPKGVGAIYIRRRPRVRVEALQSGGGQERGMRSGTVPTPLVVGLGAACEVAQQEMEYDHKRISKLSERLIQNIMKSLPDVVMNGDPKHHYPGCINLSFAYVEGESLLMALKDVALSSGSACTSASLEPSYVLRAIGTDEDLAHSSIRFGIGRFTTEEEVDYTVEKCIQHVKRLREMSPLWEMVQDGIDLKSIKWTQH	Q9Y697-1
ISD11	MGSSHHHHHHGSPTTENLYFQGHNMAASSRAQVLALYRAMLRESKRFSAYNYRTYAVRRIRDAFRENKNVKDPVEIQTLVNKAKRDLGVIRRQVHIGQLYSTDKLIIENRDMPRT	Q9HD34
ACP1	MGSDMPPLTLEGIQDRVLYVLKLYDKIDPEKLSVNSHFMKDLGLDSLDQVEIIMAMEDEFGFEIPDIDAEKLMCPQEIVDYIADKKDVYE	O14561
ISCU2	MAYHKKVVDHYENPRNVGSLDKTSKNVGTGLVGAPACGDVMKLQIQVDEKGKIVDARFKTFGCGSAIASSSLATEWVKGKTVEEALTIKNTDIAKELCLPPVKLHCSMLAEDAIKAALADYKLKQEPKKGEAEKKLEHHHHHH	Q9H1K1-1
FXN	(MHHHHHHSSGVDLGTENLYFQ)SNASGTLGHPGSLDETTYERLAEETLDSLAEFFEDLADKPYTFEDYDVSFGSGVLTVKLGGDLGTYVINKQTPNKQIWLSSPSSGPKRYDWTGKNWVYSHDGVSLHELLAAELTKALKTKLDLSSLAYSGKDA	Q16595
FDX2	MASDVVNVVFVDRSGQRIPVSGRVGDNVLHLAQRHGVDLEGACEASLACSTCHVYVSEDHLDLLPPPEEREDDMLDMAPLLQENSRLGCQIVLTPELEGAEFTLPKITRNFYVDGHVPKPH	Q6P4F2
FDXR	MGSSHHHHHHSQDPNSTQEKTPQICVVGSGPAGFYTAQHLLKHPQAHVDIYEKQPVPFGLVRFGVAPDHPEVKNVINTFTQTAHSGRCAFWGNVEVGRDVTVPELREAYHAVVLSYGAEDHRALEIPGEELPGVCSARAFVGWYNGLPENQELEPDLSCDTAVILGQGNVALDVARILLTPPEHLERTDITKAALGVLRQSRVKTVWLVGRRGPLQVAFTIKELREMIQLPGARPILDPVDFLGLQDKIKEVPRPRKRLTELLLRTATEKPGPAEAARQASASRAWGLRFFRSPQQVLPSPDGRRAAGVRLAVTRLEGVDEATRAVPTGDMEDLPCGLVLSSIGYKSRPVDPSVPFDSKLGVIPNVEGRVMDVPGLYCSGWVKRGPTGVIATTMTDSFLTGQMLLQDLKAGLLPSGPRPGYAAIQALLSSRGVRPVSFSDWEKLDAEEVARGQGTGKPREKLVDPQEMLRLLGH	P22570
ThYah1	MGHHHHHHHHHHSSGHIEGRHMLEMLKNVKDEKLINFIFLDKTPKEVFSVPGKTLLEVAHANKIDLEGACEGSLACSTCHVILDKKLYNSLEEPSDREYDLLEQAFMPCNTSRLGCQVRVDERLRNSTIKLPRATRNMAVDGFKPQPH	L7K0F4
ScYah1	MGEELKITFILKDGSQKTYEVCEGETILDIAQGHNLDMEGACGGSCACSTCHVIVDPDYYDALPEPEDDENDMLDLAYGLTETSRLGCQIKMSKDIDGIRVALPQMTRNVNNNDFS	Q12184
CtYah1	MGEELWVTFIDKEGNEHKLAVKEGDNLLDIAQAHDLEMEGACGGSCACSTCHVIVLDQEYYDRMPEPDDDENDMLDLAFGLQETSRLGCQVHMTKDLDGLRVKLPAMTRNLQASDFK	G0S5P6

Supplementary Table 2: Plasmids used for protein expression.

Plasmid	ORF	Reference
pASK-IBA43(+)_*FDX2*	*FDX2 (1-68Δ)*	ref. [1]
pET24b(+)_*ISCU2*	*ISCU2-His*$_6$ *(1-34Δ)*	ref. [2]
pETDuet1_*NFS1_ISD11*	*NFS1 (1-55Δ), His*$_6$*-Tev-ISD11*	ref. [2]
pRSFDuet1_*ACP*	*ACP (1-68Δ)*	ref. [2]
pMCSG7_*FXN*	*His*$_6$*-Tev-FXN (1-80Δ)*	ref. [2]
pETDuet1_*FDXR*	*His*$_6$*-FDXR (1-32Δ)*	ref. [3]
pASK-IBA43(+)_*FDX2-C12*	*FDX2 (1-68Δ, 175-186Δ)*	ref. [4]
pASK-IBA43(+)_*FDX2-C10*	*FDX2 (1-68Δ, 177-186Δ)*	this work
pASK-IBA43(+)_*FDX2-C5*	*FDX2 (1-68Δ, 182-186Δ)*	this work
pASK-IBA43(+)_*FDX2-C1*	*FDX2 (1-68Δ, 186Δ)*	this work
pASK-IBA43(+)_*FDX2-N175A*	*FDX2 (1-68Δ, N175A)*	this work
pASK-IBA43(+)_*FDX2-DD*	*FDX2 (1-68Δ, D137A, D138A)*	this work
pASK-IBA43(+)_*FDX2-EASL*	*FDX2 (1-68Δ, E109G, A110G, L112C)*	this work
pETDuet_NFS1-RA_ISD11	*NFS1 (R271A, R272A, R273A, R275A, R277A)*	this work
pET16b_*ThYah1*	*His*$_{10}$*-ThYah1*	ref. [5]
pET15b_*ScYah1*	*ScYah1 (1-57Δ)*	ref. [1]
pETDuet1_*CtYah1*	*CtYah1 (1-86 Δ)*	ref. [6]

Supplementary Table 3: Data collection parameters, processing parameters and model validation statistics for the $(NIAUF)_2$ dataset.

	$(NIAUF)_2$ consensus map	$(NIAUF)_2$ FDX2-bound proximal	$(NIAUF)_2$ FDX2-bound distal
Map EMDB ID	**EMD-19355**	**EMD-19356**	**EMD-19357**
**Data collection**			
Microscope	Krios G4	Krios G4	Krios G4
Camera	Falcon 4	Falcon 4	Falcon 4
Voltage (kV)	300	300	300
Nominal magnification	215,000x	215,000x	215,000x
Calibrated pixel size (Å)	0.573	0.573	0.573
Dose (e^-/Å^2)	80	80	80
Number of frames per image	952	952	952
Defocus range (µm)	-2.2 - -0.8	-2.2 - -0.8	-2.2 - -0.8
**Image processing**			
Motion correction software	MotionCor2	MotionCor2	MotionCor2
CTF estimation software	CTFFIND4	CTFFIND4	CTFFIND4
Particle selection software	Topaz	Topaz	Topaz
Final micrographs (no.)	8497	8497	8497
Initial particle images (no.)	903,238	903,238	903,238
Final particle images (no.)	363,652	449,255 (symmetry expanded)	88,788 (symmetry expanded)
Symmetry applied	C2	C1	C1
Map sharpening B-factor (Å^2)	-58.2	*	*
Final resolution (Å)	2.03	2.26	2.52
FSC threshold	0.143	0.143	0.143
Model PDB ID		**8RMC**	**8RMD**
**Refinement**			
Modeling software		Coot, PHENIX	Coot, PHENIX
Protein residues		1482	1461
Water		400	290
Ligands		8Q1, FE2, FES, PLP	8Q1, FE2, FES, PLP
**Validation**			
MolProbity score		1.35	1.48
Clash score		4.72	6.25
Ramachandran plot (%)			
Outliers		0.00	0.00
Allowed		1.50	1.66
Favored		98.50	98.34
Rotamer outliers (%)		1.40	1.51
Cβ outliers (%)		0.07	0.00
Peptide plane (%)			
Cis proline/general		3.3/0.0	3.5/0.0
Twisted proline/general		0.0/0.0	0.0/0.0
CaBLAM outliers (%)		0.55	0.70

* the primary map was density modified with *phenix.resolve_cryo_em*

Supplementary Table 4: Data collection parameters, processing parameters and model validation statistics for the $(NIAUXF)_2$ turnover datasets.

	$(NIAUXF)_2$ turnover, consensus map	$(NIAUXF)_2$ turnover, FXN-bound	$(NIAUXF)_2$ turnover, FDX2-bound proximal	$(NIAUXF)_2$ turnover, FDX2-bound distal
Map EMDB ID	**EMD-19358**	**EMD-19359**	**EMD-19360**	**EMD-19361**
**Data collection**				
Microscope	Krios G4	Krios G4	Krios G4	Krios G4
Camera	Falcon 4	Falcon 4	Falcon 4	Falcon 4
Voltage (kV)	300	300	300	300
Nominal magnification	215,000x	215,000x	215,000x	215,000x
Calibrated pixel size (Å)	0.573	0.573	0.573	0.573
Dose (e^-/Å^2)	80	80	80	80
Number of frames per image	987 /1,078	987 / 1,078	987 / 1,078	987 / 1,078
Defocus range (µm)	-2.2 - -0.8	-2.2 - -0.8	-2.2 - -0.8	-2.2 - -0.8
**Image processing**				
Motion correction software	MotionCor2	MotionCor2	MotionCor2	MotionCor2
CTF estimation software	CTFFIND4	CTFFIND4	CTFFIND4	CTFFIND4
Particle selection software	Topaz	Topaz	Topaz	Topaz
Final micrographs (no.)	17,891	17,891	17,891	17,891
Initial particle images (no.)	1,894,025	1,894,025	1,894,025	1,894,025
Final particle images (no.)	731,041	276,036 (symmetry expanded)	195,329 (symmetry expanded)	326,204 (symmetry expanded)
Symmetry applied	C2	C1	C1	C1
Map sharpening B-Factor (Å^2)	-68.2	-60	-58.2	-69.1
Final resolution (Å)	2.09	2.49	2.33	2.46
FSC threshold	0.143	0.143	0.143	0.143
Model PDB ID		**8RME**	**8RMF**	**8RMG**
**Refinement**				
Modeling software		Coot, PHENIX	Coot, PHENIX	Coot, PHENIX
Protein residues		1466	1466	1463
Water		157	253	158
Ligands		8Q1, FE2, PLP	8Q1, FE2, FES, PLP	8Q1, FE2, FES, PLP
**Validation**				
MolProbity score		1.54	1.35	1.53
Clash score		5.41	5.74	6.32
Ramachandran plot (%)				
Outliers		0.00	0.00	0.00
Allowed		2.35	2.14	1.52
Favored		97.65	97.86	98.48
Rotamer outliers (%)		1.67	0.87	1.75
Cβ outliers (%)		0.00	0.07	0.15
Peptide plane (%)				
Cis proline/general		3.7/0.0	3.4/0.0	3.5/0.0
Twisted proline/general		0.0/0.0	0.0/0.0	0.0/0.0
CaBLAM outliers (%)		0.49	0.77	0.70

Supplementary References

1 Webert, H. *et al.* Functional reconstitution of mitochondrial Fe/S cluster synthesis on Isu1 reveals the involvement of ferredoxin. *Nat Commun* **5**, 5013 (2014). https://doi.org/10.1038/ncomms6013

2 Freibert, S.-A. *et al.* N-terminal tyrosine of ISCU2 triggers [2Fe-2S] cluster synthesis by ISCU2 dimerization. *Nat Commun* **12**, 6902 (2021). https://doi.org/10.1038/s41467-021-27122-w

3 Sheftel, A. D. *et al.* Humans possess two mitochondrial ferredoxins, Fdx1 and Fdx2, with distinct roles in steroidogenesis, heme, and Fe/S cluster biosynthesis. *Proc Natl Acad Sci U S A* **107**, 11775-11780 (2010). https://doi.org/10.1073/pnas.1004250107

4 Schulz, V. *et al.* Functional spectrum and specificity of mitochondrial ferredoxins FDX1 and FDX2. *Nat Chem Biol* **19**, 206-217 (2023). https://doi.org/10.1038/s41589-022-01159-4

5 Freibert, S.-A. *et al.* Evolutionary conservation and in vitro reconstitution of microsporidian iron–sulfur cluster biosynthesis. *Nat Commun* **8**, 13932 (2017). https://doi.org/10.1038/ncomms13932

6 Boniecki, M. T., Freibert, S. A., Mühlenhoff, U., Lill, R. & Cygler, M. Structure and functional dynamics of the mitochondrial Fe/S cluster synthesis complex. *Nat Commun* **8**, 1287 (2017). https://doi.org/10.1038/s41467-017-01497-1
