# Supplementary material for: Two-stage binding of mitochondrial ferredoxin-2 to the core iron-sulfur cluster assembly complex
Source: Nat Commun. 2024 Dec 4;15:10559. doi: 10.1038/s41467-024-54585-4 (PMC11618653; doi:10.1038/s41467-024-54585-4)
Supplement: Supplementary file 2 — Description of Additional Supplementary Files [file 41467_2024_54585_MOESM2_ESM.pdf]

## Description of Additional Supplementary Files

**File name: Supplementary Movie 1**
**Description:** Morph between the distal (PDB 8RMD) and proximal (PDB 8RMC) $(NIAUF)_2$ conformations, showing the rotation of FDX2 helix F along the NFS1 arginine patch, the movement of the ISCU2 Cys69-loop, and the interaction of the C terminus of FDX2 with NFS1 in the proximal conformation.
