# Supplementary material for: Two-stage binding of mitochondrial ferredoxin-2 to the core iron-sulfur cluster assembly complex
Source: Nat Commun. 2024 Dec 4;15:10559. doi: 10.1038/s41467-024-54585-4 (PMC11618653; doi:10.1038/s41467-024-54585-4)
Supplement: Supplementary file 4 — Reporting Summary [file 41467_2024_54585_MOESM4_ESM.pdf]

nature portfolio

Corresponding author(s): Bonnie Murphy, Roland Lill

Last updated by author(s): 24/10/2024

Reporting Summary

Nature Portfolio wishes to improve the reproducibility of the work that we publish. This form provides structure for consistency and transparency in reporting. For further information on Nature Portfolio policies, see our Editorial Policies and the Editorial Policy Checklist.

Statistics

For all statistical analyses, confirm that the following items are present in the figure legend, table legend, main text, or Methods section.

n/a	Confirmed	
☐	☒	The exact sample size (*n*) for each experimental group/condition, given as a discrete number and unit of measurement
☐	☒	A statement on whether measurements were taken from distinct samples or whether the same sample was measured repeatedly
☒	☐	The statistical test(s) used AND whether they are one- or two-sided<br>*Only common tests should be described solely by name; describe more complex techniques in the Methods section.*
☒	☐	A description of all covariates tested
☒	☐	A description of any assumptions or corrections, such as tests of normality and adjustment for multiple comparisons
☐	☒	A full description of the statistical parameters including central tendency (e.g. means) or other basic estimates (e.g. regression coefficient) AND variation (e.g. standard deviation) or associated estimates of uncertainty (e.g. confidence intervals)
☒	☐	For null hypothesis testing, the test statistic (e.g. *F*, *t*, *r*) with confidence intervals, effect sizes, degrees of freedom and *P* value noted<br>*Give P values as exact values whenever suitable.*
☒	☐	For Bayesian analysis, information on the choice of priors and Markov chain Monte Carlo settings
☒	☐	For hierarchical and complex designs, identification of the appropriate level for tests and full reporting of outcomes
☒	☐	Estimates of effect sizes (e.g. Cohen's *d*, Pearson's *r*), indicating how they were calculated

Our web collection on statistics for biologists contains articles on many of the points above.

Software and code

Policy information about availability of computer code

Data collection	EPU 3.1, EPU 3.3
Data analysis	cryoSPARC live, RELION-4, MotionCor2, CTFFind4, cryoSPARC v4.1.2, pyem, PHENIX 1.20.1-4487, Coot-0.9, Prism 5

For manuscripts utilizing custom algorithms or software that are central to the research but not yet described in published literature, software must be made available to editors and reviewers. We strongly encourage code deposition in a community repository (e.g. GitHub). See the Nature Portfolio guidelines for submitting code & software for further information.

Data

Policy information about availability of data

All manuscripts must include a data availability statement. This statement should provide the following information, where applicable:

- Accession codes, unique identifiers, or web links for publicly available datasets
- A description of any restrictions on data availability
- For clinical datasets or third party data, please ensure that the statement adheres to our policy

Cryo-EM maps and atomic models from the (NIAUF)2 dataset were deposited to the Electron Microscopy Data Bank and the Protein Data Bank under the accession codes EMD-19355 [https://www.ebi.ac.uk/emdb/EMD-19355] ((NIAUF)2 consensus map), EMD-19356 [https://www.ebi.ac.uk/emdb/EMD-19356] and PDB 8RMC [http://doi.org/10.2210/pdb8RMC/pdb] (FDX2-bound proximal), EMD-19357 [https://www.ebi.ac.uk/emdb/EMD-19357] and PDB 8RMD [http://doi.org/10.2210/pdb8RMD/pdb] (FDX2-bound distal). Cryo-EM maps and atomic models originating from the (NIAUXF)2 turnover datasets are accessible under EMD-19358 [https://

www.ebi.ac.uk/emdb/EMD-19358] ((NIAUXF)2 turnover, consensus map), EMD-19359 [https://www.ebi.ac.uk/emdb/EMD-19359] and PDB 8RME [http://doi.org/10.2210/pdb8RME/pdb] ((NIAUXF)2 turnover, FXN-bound), EMD-19360 [https://www.ebi.ac.uk/emdb/EMD-19360] and PDB 8RMF [http://doi.org/10.2210/pdb8RMF/pdb] ((NIAUXF)2 turnover, FDX2-bound proximal), EMD 19361 [https://www.ebi.ac.uk/emdb/EMD-19361] and PDB 8RMG [http://doi.org/10.2210/pdb8RMG/pdb] ((NIAUXF)2 turnover, FDX2-bound distal). Source data are provided with this paper.

Research involving human participants, their data, or biological material

Policy information about studies with human participants or human data. See also policy information about sex, gender (identity/presentation), and sexual orientation and race, ethnicity and racism.

Reporting on sex and gender	Not applicable as the study did not involve human participants, their data or biological material
Reporting on race, ethnicity, or other socially relevant groupings	Not applicable as the study did not involve human participants, their data or biological material
Population characteristics	Not applicable as the study did not involve human participants, their data or biological material
Recruitment	Not applicable as the study did not involve human participants, their data or biological material
Ethics oversight	Not applicable as the study did not involve human participants, their data or biological material

Note that full information on the approval of the study protocol must also be provided in the manuscript.

Field-specific reporting

Please select the one below that is the best fit for your research. If you are not sure, read the appropriate sections before making your selection.

☒ Life sciences ☐ Behavioural & social sciences ☐ Ecological, evolutionary & environmental sciences

For a reference copy of the document with all sections, see nature.com/documents/nr-reporting-summary-flat.pdf

Life sciences study design

All studies must disclose on these points even when the disclosure is negative.

Sample size	The number of images collected was determined by availability of electron microscope time. Sample size (n) in biochemical studies was not predetermined. For all measurements the sample size is stated and represents technical repetitions of the respective experiment.
Data exclusions	Particle images were excluded by 2- and 3-dimensional classification, with classes selected by visual inspection, according to established practice in the field. No data from the biochemical analyses were excluded.
Replication	All attempts at replication were successful. The number of replications is stated for each experiment individually in the figure legends. The biochemical experiments were reproducible with newly prepared biological material (proteins or protein complexes).
Randomization	Particle images were randomly assigned to data half-sets automatically by the data processing software. n/a for the biochemical experiments, because randomization is not needed for this type of experiments.
Blinding	Blinding is not relevant to the study. For cryo-EM analysis, experimenter intervention is required for the assessment of image / map quality and interpretation, eg. for 2D and 3D class selection and atomic model building, according to established practice in the field. For biochemical assays, the result obtained is an objective measurement not dependent upon the experimenter's assessment, therefore blinding is not relevant to such a study.

Reporting for specific materials, systems and methods

We require information from authors about some types of materials, experimental systems and methods used in many studies. Here, indicate whether each material, system or method listed is relevant to your study. If you are not sure if a list item applies to your research, read the appropriate section before selecting a response.

Materials & experimental systems

n/a	Involved in the study
☒	☐ Antibodies
☒	☐ Eukaryotic cell lines
☒	☐ Palaeontology and archaeology
☒	☐ Animals and other organisms
☒	☐ Clinical data
☒	☐ Dual use research of concern
☒	☐ Plants

Methods

n/a	Involved in the study
☒	☐ ChIP-seq
☒	☐ Flow cytometry
☒	☐ MRI-based neuroimaging

Plants

Seed stocks	Not applicable as the study did not involve plants
Novel plant genotypes	Not applicable as the study did not involve plants
Authentication	Not applicable as the study did not involve plants
